# Augmented Endothelial-Specific L-Arginine Transport Blunts the Contribution of the Sympathetic Nervous System to Obesity Induced Hypertension in Mice

**DOI:** 10.1371/journal.pone.0131424

**Published:** 2015-07-17

**Authors:** Niwanthi W. Rajapakse, Florian Karim, Roger G. Evans, David M. Kaye, Geoffrey A. Head

**Affiliations:** 1 Baker IDI Heart and Diabetes Institute, Melbourne, Australia; 2 Department of Physiology, Monash University, Melbourne, Australia; East Tennessee State University, UNITED STATES

## Abstract

Augmenting endothelial specific transport of the nitric oxide precursor L-arginine via cationic amino acid transporter-1 (CAT1) can prevent obesity related hypertension. We tested the hypotheses that CAT1 overexpression prevents obesity-induced hypertension by buffering the influence of the sympathetic nervous system (SNS) on the maintenance of arterial pressure and by buffering pressor responses to stress. Wild type (WT; n=13) and CAT1 overexpressing mice (CAT+; n=13) were fed a normal or a high fat diet for 20 weeks. Mice fed a high fat diet were returned to the control diet before experiments commenced. Baseline mean arterial pressure (MAP) and effects of restraint-, shaker- and almond feeding-stress and ganglionic blockade (pentolinium; 5 mg/kg; i.p.) on MAP were determined in conscious mice. Fat feeding increased body weight to a similar extent in WT and CAT+ but MAP was greater only in WT compared to appropriate controls (by 29%). The depressor response to pentolinium was 65% greater in obese WT than lean WT (*P* < 0.001), but was similar in obese and lean CAT+ (*P* = 0.65). In lean WT and CAT+, pressor responses to shaker and feeding stress, but not restraint stress, were less in the latter genotype compared to the former (*P* ≤ 0.001). Pressor responses to shaker and feeding stress were less in obese WT than lean WT (*P* ≤ 0.001), but similar in obese and lean CAT+. The increase in MAP in response to restraint stress was less in obese WT (22 ± 2%), but greater in obese CAT+ (37 ± 2%), when compared to respective lean WT (31 ± 3%) and lean CAT+ controls (27 ± 2%; *P* ≤ 0.02). We conclude that CAT1 overexpression prevents obesity-induced hypertension by reducing the influence of the SNS on the maintenance of arterial pressure but not by buffering pressor responses to stress.

## Introduction

The role of nitric oxide (NO) in long term regulation of arterial pressure is now well established [1, 2]. L-arginine is the substrate for NO formation and cationic amino acid transporter-1 (CAT-1) is the predominant L-arginine transporter expressed in endothelial cells. Importantly, NO bioavailability depends on the cellular transport of L-arginine [1–3]. We recently made the startling discovery that augmenting endothelial specific transport of L-arginine via CAT-1 can increase NO bioavailability and prevent obesity-induced hypertension in mice [4]. These data indicate that endothelial CAT1 overexpression and subsequent increases in NO bioavailability can block the mechanistic link between obesity and hypertension. In this context, there is compelling evidence that augmented activity of the sympathetic nervous system (SNS) plays a central role in the development of obesity-induced hypertension [5–7]. There is evidence that NO can buffer neurally mediated vasoconstriction within the periphery [8]. Importantly, NO can also reduce sympathetic outflow [9]. Therefore, endothelial CAT1 overexpression and subsequent increases in NO bioavailability may prevent obesity-induced hypertension, at least in part, by reducing the sympathetic contribution to hypertension. The present study was designed to test the hypothesis that increasing endothelial specific L-arginine transport prevents obesity induced hypertension by reducing the sympathetic contribution to hypertension. Therefore, we compared the depressor responses to ganglionic blockade in lean and obese mice with endothelial overexpression of CAT1 (CAT+ mice) with those in wild-type mice (WT).

Augmented pressor responses to psychological stress [6] are also considered a risk factor in the development of obesity-related hypertension [10]. In this context, there is evidence that NO can blunt neurally mediated vasoconstriction during stress in humans of normal weight [11]. NO bioavailability, including that within the brain, is reduced in obesity [4, 12] which may contribute to impaired blood pressure regulation during stress. Indeed, multiple studies have demonstrated that increased adiposity is associated with impaired blood pressure regulation during psychological stress [13, 14]. For example, stressful stimuli induced lesser increases in noradrenaline release in Obese Zucker rats compared to controls [15]. However, there is not universal agreement on this point, since stress induced greater pressor responses in a small number of young obese subjects compared to non-obese subjects [14]. The pressor response to airjet stress was also found to be greater in obese rats compared to controls [16] but this was due to ineffective vasodilatation via β-adrenergic mechanisms and not related to activation of sympathetic vasomotor tone [16]. Thus, studies of the impact of obesity on the pressor responses to stress have yielded disparate results. Consequently, we hypothesised that the impact of obesity on stress varies according to the specific stressor. Therefore, we subjected lean and obese WT and CAT+ mice to three different stressors that operate through distinct neural pathways [17, 18]. We hypothesised that endothelial CAT1 overexpression can blunt pressor responses to stress.

## Methods

### Animals

CAT+ mice were generated as previously described [19]. WT C57BL/6 mice (*n* = 13) and CAT+ mice (*n* = 13) were fed either a normal fat diet or a high fat diet (43% fat, SF04-001;Speciality Feeds, Perth, WA, Australia) for a period of 20 weeks. The dietary intervention commenced immediately after weaning and mice were allowed access to food and water *ad libitum*. Mice on a high fat diet were returned to the control diet seventeen days before experiments commenced, to ensure we could attribute differences between the groups to their adiposity rather than their diet. All mice were housed on a 12:12 hour light-dark cycle (6:00 AM to 6:00 PM light). This study was approved by the Alfred Medical Research Education Precinct Animal Ethics Committee and all experiments were conducted in accordance with the Australian Code of Practice for the Care and Use of Animals for Scientific Purposes. *In vivo* data from the cohort of animals used in the current study have been reported elsewhere [4]. However, none of the current data have previously been published.

### Surgical Preparation

Radiotelemetry probes were implanted in mice under 2.5–5.5% v/v isoflurane anaesthesia (Isoflo, Abbott Australasia Pty Ltd, Botany, NSW, Australia). The tip of the catheter was inserted to the left carotid artery and the body of the probe was placed in the subcutaneous pocket as close to the right hind-limb as possible [20]. Experiments commenced 20 days after implantation of telemetry probes.

### Data Acquisition

Pulsatile arterial pressure and gross locomotor activity were monitored and were sampled at 1000 Hz using an analog-to-digital data acquisition card. Heart rate (HR) and beat-to-beat mean arterial pressure (MAP) were detected online and analysed as previously described [17].

#### Assessment of Cardiovascular Reactivity in Response to Aversive and Non-Aversive Stress Stimuli

A series of non-aversive (eating) and aversive (restraint, shaker) stimuli were used to induce behavioural stress during the period when mice were inactive [21]. These stimuli have been previously shown to increase arterial pressure in mice [21, 22].

Prior to performing the stress tests, baseline MAP, HR and locomotor activity were recorded for at least 1 hour. During this period, all mice were left undisturbed. After a stable baseline was obtained, stress tests (almond feed, shaker stress, restraint) were conducted in a random order. Each stress test was of 5 min duration. Sixty to ninety minutes were allowed between tests to allow mice to recover completely between stress stimuli.

Restraint stress was induced by guiding the mouse into a cylindrical plexiglass restrainer and restricting the movement of the mouse without causing any physical pressure. The mouse was restrained for a 5 minute period before being released back to its home cage.

Feeding stress was initiated by providing mice with approximately 1/3 to 1/2 of an almond. MAP and HR were recorded for a 5-minute period which commenced immediately after the mouse started eating. Only data from mice that fed for a period of at least 5 min were included in the final analysis.

Shaker stress was initiated by placing the mouse cage and the telemetric receiver plate on an orbital mixing machine at a speed of 90 rotations/min (RATEK orbital mixer, Ratek Instruments Pty Ltd, Victoria, Australia). After 5 min, the orbital mixer was switched off. The home cage containing the mouse was then returned to its original position.

#### Effects of the ganglionic blocker pentolinium

Twenty four hours after responses to stress stimuli had been recorded, responses of MAP, HR and locomotor activity were recorded for 30 min prior to and 30 min after administration of the ganglionic blocker, pentolinium (5 mg/kg i.p.; *n* = 5–7; Sigma, Sydney, Australia). This test was performed during the active phase of the circadian rhythm. Seventy two hours after pentolinium administration, mice were humanely killed by overdose with pentobarbitone (163 mg/kg).

### Statistical analysis

Data are presented as mean ± SEM and mean difference ± SE of the difference. An unpaired *t*-test was used for comparisons of body weight after dietary intervention. A two factor mixed model repeated measure ANOVA (adiposity and genotype) was used to determine (i) the effects of adiposity (*P*_Adiposity_) (ii) the effects of genotype (*P*_Genotype_) and (iii) whether the effects of adiposity on baseline levels of arterial pressure and responses of arterial pressure to stress stimuli and the ganglionic blocker pentolinium differed between the two genotypes of mice (*P*_Adiposity_*_Genotype_). Additional non-orthogonal dichotomous comparisons were made to determine the effects of diet within the same genotype (*P*_Adiposity within genotype_). Accordingly, familywise error (increased risk of Type 1 error) arising from comparisons between lean WT *vs* lean CAT+ mice and obese *vs* lean mice within each genotype was accounted for by a Bonferroni adjustment. Violations of sphericity from relatedness of repeated measures were corrected by the Greenhouse-Geisser method. Two-tailed *P* ≤ 0.05 was considered statistically significant.

## Results

### Influence of adiposity on baseline levels of variables

**Fig 1:**
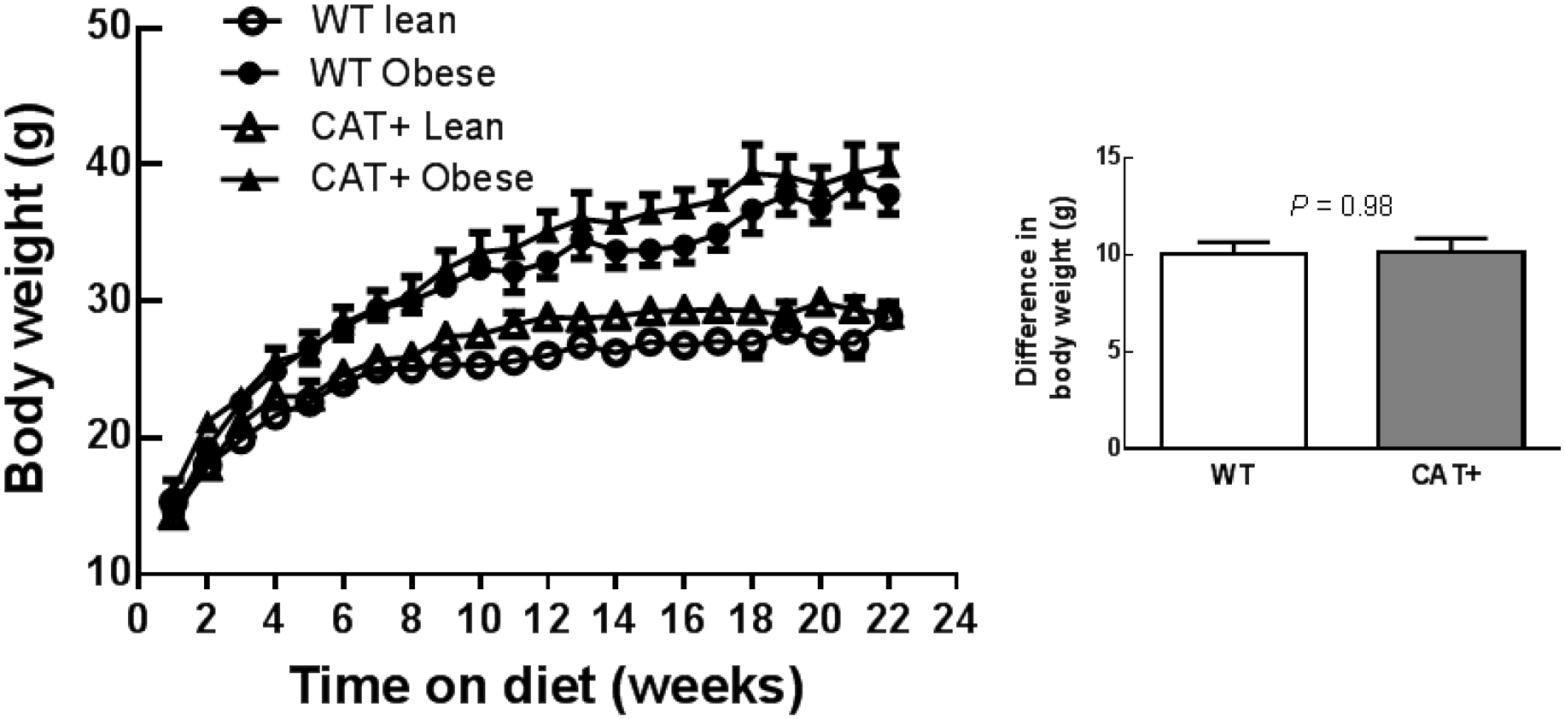
(A) Weight gain in WT and CAT+ mice at the end of the dietary intervention. Data are mean ± SEM (*n* = 6–7). (B) Difference in body weight between lean and obese mice at the end of the 157 day diet protocol. Data are mean difference ± SED. The *P* value was derived from an unpaired *t*-test.

Fat feeding increased body weight in both WT and CAT+ mice (*P*_Adiposity within genotype_ ≤ 0.001; Fig 1). Of note, body weight at the end of the dietary intervention was not significantly different in obese WT and CAT+ mice (*P* = 0.55). MAP during the 1-h period immediately prior to performing stress tests was 29% greater in obese WT compared to lean WT (*P*_Adiposity within genotype_ = 0.01; Table 1). In contrast, MAP was similar between lean and obese CAT+ mice (*P*_Adiposity within genotype_ = 0.52; *P*_Adiposity_*_Genotype_ = 0.02; Table 1). HR and locomotor activity did not differ significantly between lean and obese mice, regardless of their genotype (*P*_Adiposity within genotype_ ≥ 0.43; Table 1).

**Table 1:** Baseline levels of variables in wild type and cationic amino acid transporter-1 overexpressing mice in the 1-h periods immediately prior to stress tests.

	Lean	Obese
**MAP (mmHg)**		
WT	84 ± 5	108 ± 5*
CAT+	89 ± 3	93 ± 5
**HR (b.p.m)**		
WT	590 ± 41	634 ± 42
CAT+	536 ± 32	587 ± 49
**Locomotor activity (units)**		
WT	1.12 ± 0.33	0.82 ± 0.43
CAT+	0.90 ± 0.14	0.83 ± 0.32

Data are mean ± SEM (*n* = 5–7).

* *P* ≤ 0.01 for obese compared with lean mice within the same genotype. *P* values were derived from un-paired *t*-tests. WT = wild type mice, CAT+ = endothelial CAT1 overexpressing mice, MAP = mean arterial pressure, HR = heart rate.

### Influence of adiposity on responses of MAP to the ganglionic blocker pentolinium

**Fig 2:**
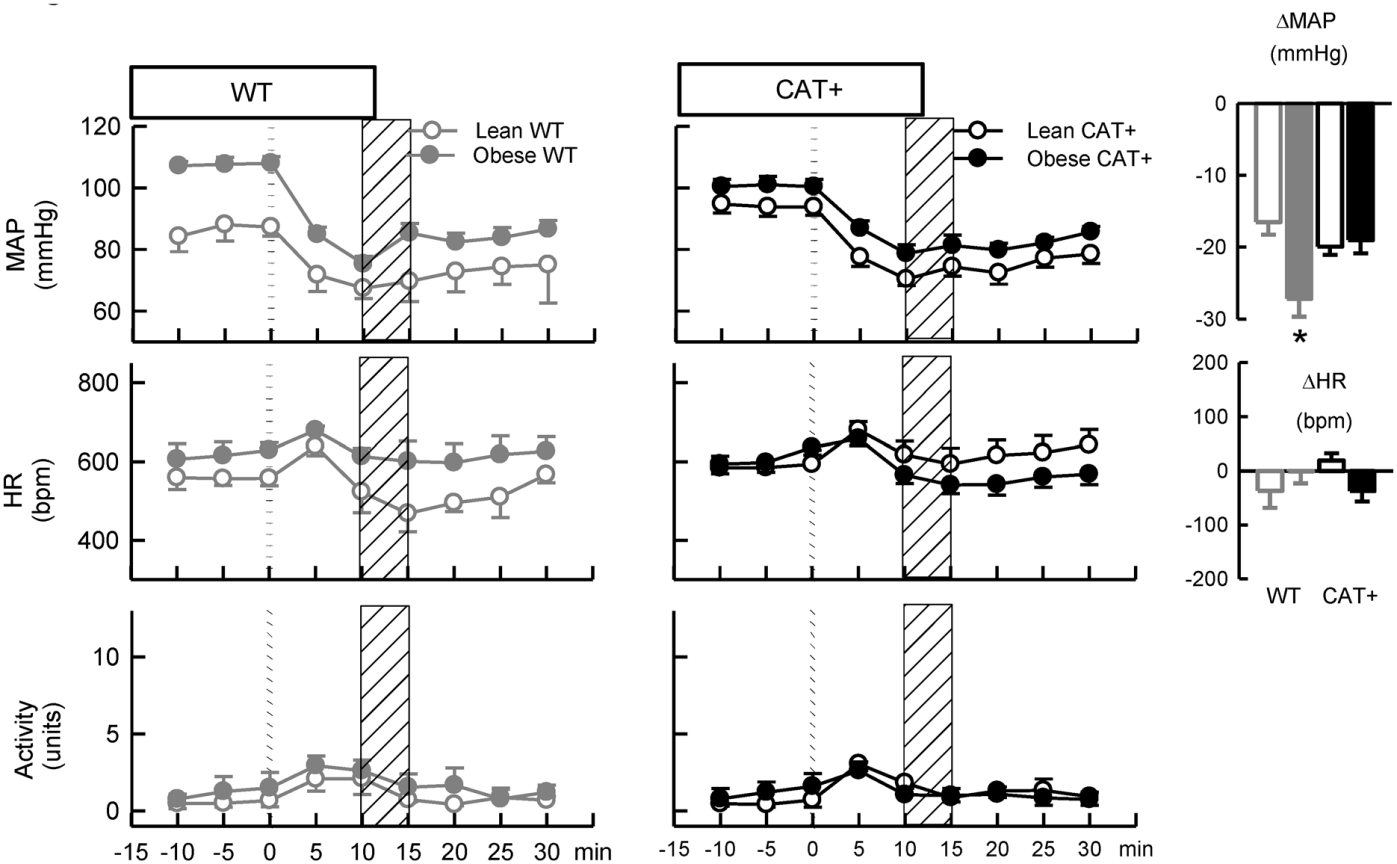
Responses to the ganglionic blocker pentolinium in lean and obese wild type and endothelial CAT1 overexpressing mice. Left and middle panels: Each symbol represents mean values averaged across a 5 min period. The dotted line represents the time point at which pentolinium (5 mg/kg, i.p.) was administered and the shaded areas represent the period analysed for between-group comparisons of the effects of pentolinium. Data are mean ± SEM. Right panel: Histograms represent the average of absolute changes in variables in response to pentolinium. Data are mean difference ± SE of the difference (*n* = 5–7).* *P* < 0.05 for obese *vs* lean mice within the same genotype. WT = wild type mice, CAT+ = endothelial CAT1 overexpressing mice, MAP = mean arterial pressure, HR = heart rate.

The depressor response to pentolinium was similar in lean WT and CAT+ mice (*P*_*Genotype*_ = 0.14; Fig 2). The absolute reduction in MAP in response to pentolinium was 65% greater in obese WT mice compared to lean WT mice (*P*_Adiposity within genotype_ < 0.001; Fig 2). In contrast, depressor responses to pentolinium did not differ significantly between lean and obese CAT+ mice (*P*_Adiposity within genotype_ = 0.65; *P*_Adiposity_*_Genotype_ = 0.003). After ganglionic blockade, MAP was similar in obese WT and CAT+ mice (*P* = 0.78; Fig 2).

### Responses to behavioural stress in lean mice

**Fig 3:**
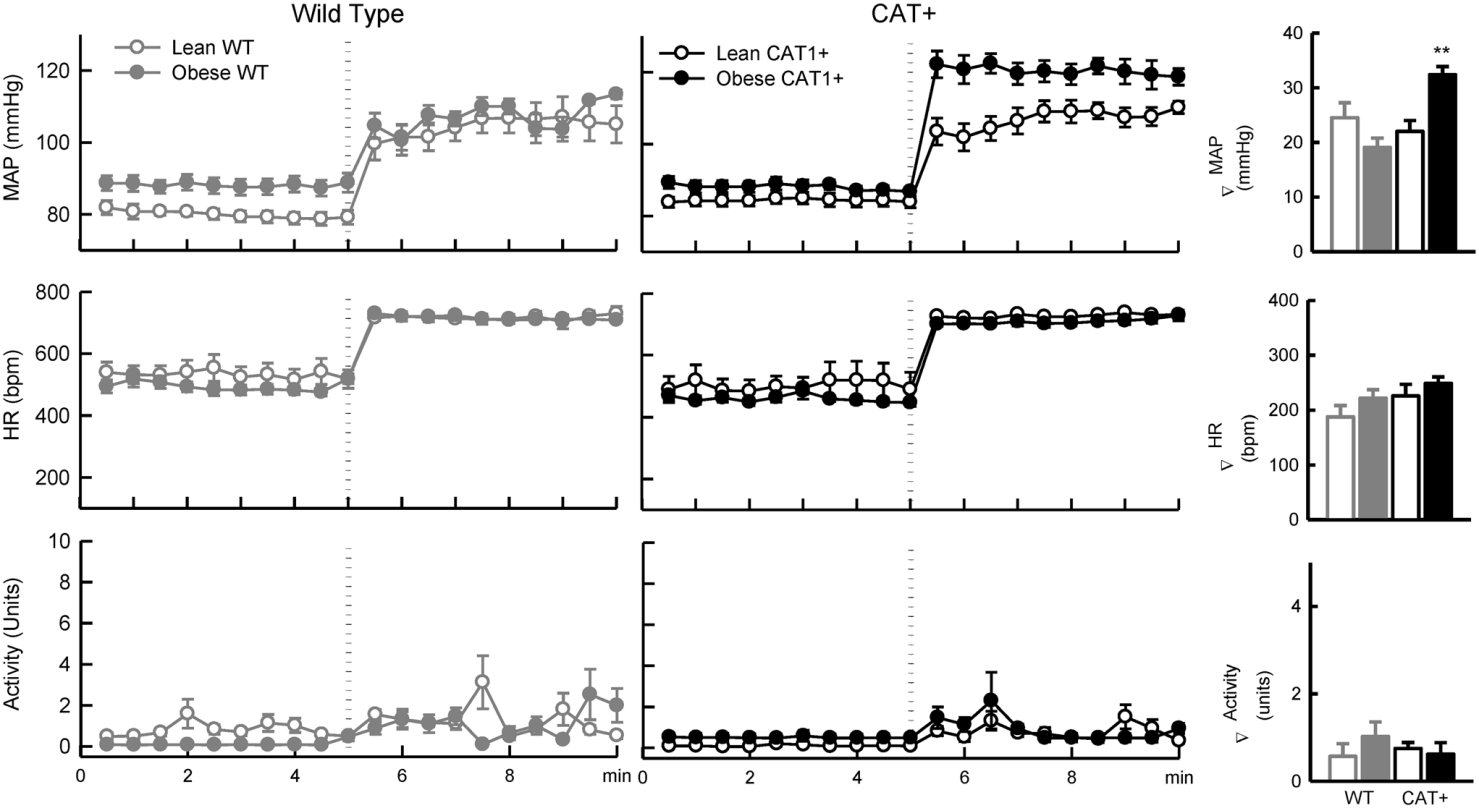
Responses to restraint stress in lean and obese mice. Left and middle panels: Each symbol represents mean values averaged across a 30-s period. Dotted lines represent the time point at which the stress stimulus was applied to mice. Data are mean ± SEM. Right panel: Bar graphs represent absolute changes from baseline in MAP, HR and locomotor activity in response to 5-min restraint stress in mice fed a normal (open bars) or a high fat diet (closed bars). Data are mean difference ± SE of the difference (*n* = 5–7). ** *P* < 0.01 for obese *vs* lean mice within the same genotype. Abbreviations are as for Fig 2.

**Fig 4:**
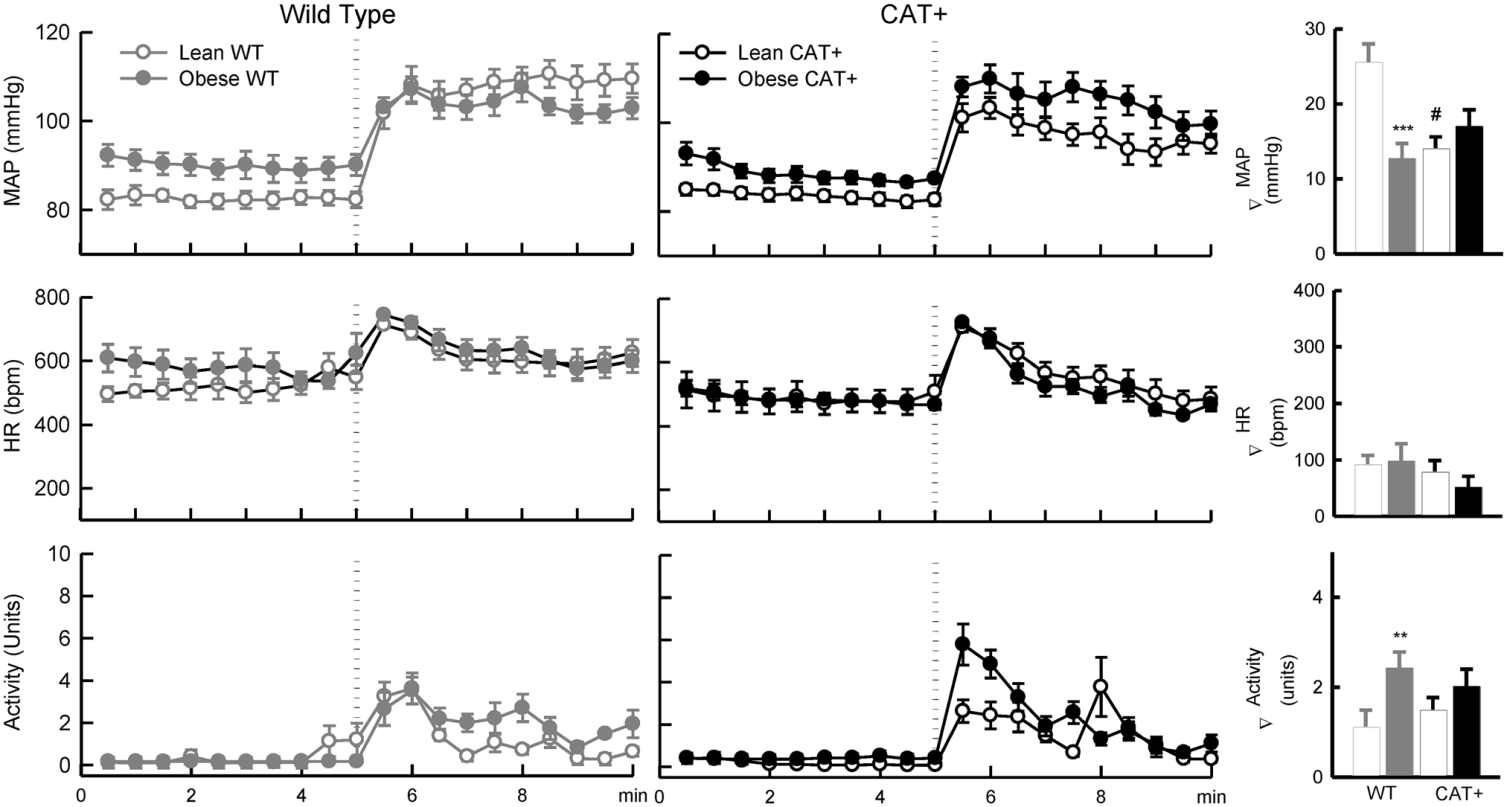
Responses to shaker stress in lean and obese mice. Left and middle panels: Each symbol represents mean values averaged across a 30-s period. Dotted line represents the time point at which stress stimulus was applied to mice. Data are mean ± SEM. Right panel: Bar graphs represent absolute changes from baseline in MAP, HR and locomotor activity in response to 5-min shaker stress in mice fed a normal (open bars) or a high fat diet (closed bars) (*n* = 5–7). Data are mean difference ± SE of the difference (*n* = 5–7). *** *P* < 0.001 and ** *P* < 0.01 for obese *vs* lean mice within the same genotype. # *P* < 0.001 lean CAT+ mice *vs* lean WT mice. Abbreviations are as for Fig 2.

**Fig 5:**
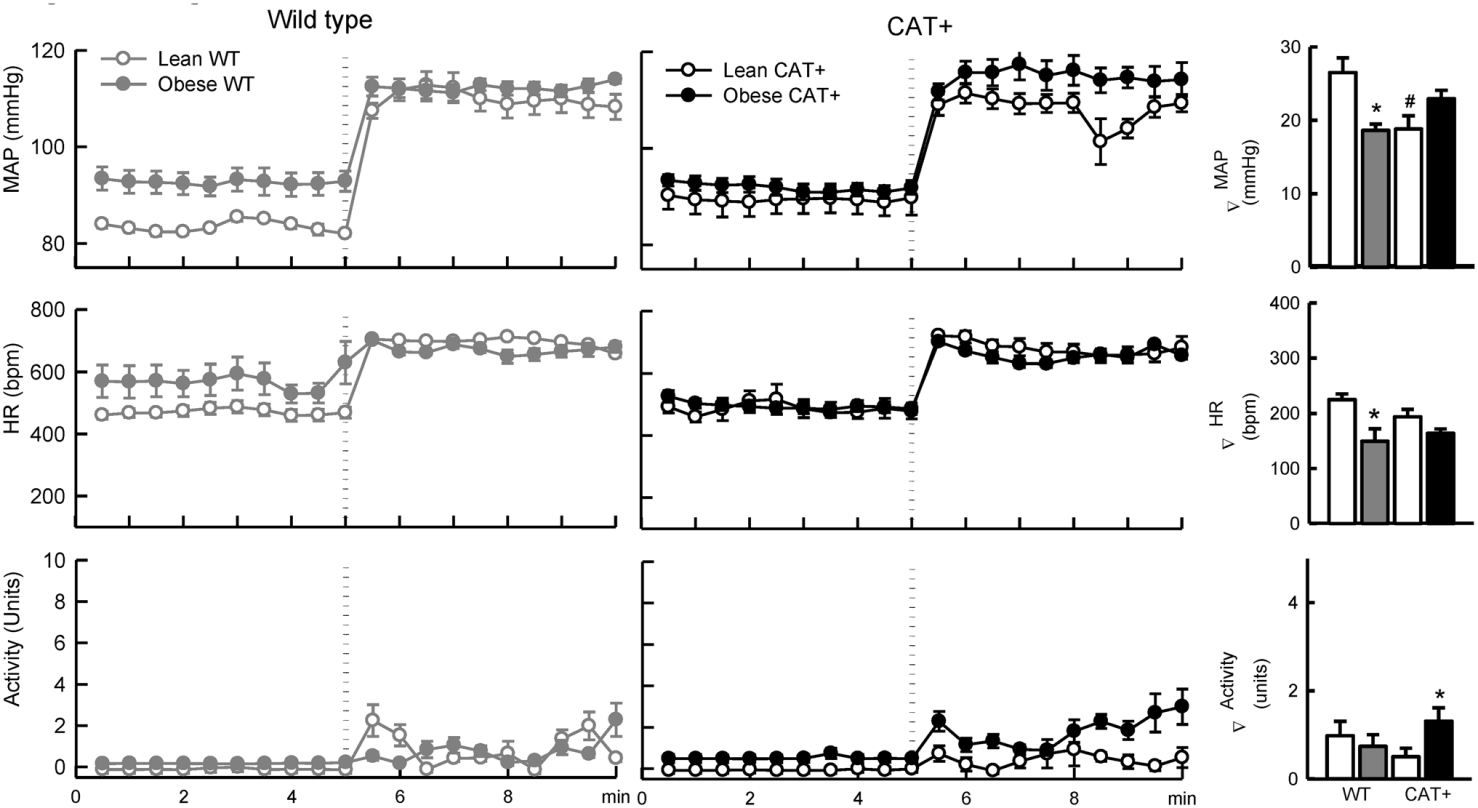
Responses to almond feeding stress in lean and obese mice. Left and middle panels: Each symbol represents mean values averaged across a 30-s period. Dotted line represents the time point at which stress stimulus was applied to mice. Data are mean ± SEM. Right panel: Bar graphs represent absolute changes from baseline in MAP, HR and locomotor activity in response to 5-min feeding stress in mice fed a normal (open bars) or a high fat diet (closed bars). Data are mean difference ± SE of the difference (*n* = 5–7). * *P* < 0.05 for obese *vs* lean mice within the same genotype. # *P* < 0.01 for lean CAT+ mice *vs* lean WT mice. Abbreviations are as for Fig 2.

In lean mice, pressor responses to shaker and almond feeding, but not restraint stress, were less in CAT+ than WT (Figs 3, 4 and 5). In lean WT mice, MAP increased by 32 ± 3% and 32 ± 2% in response to shaker and feeding, respectively. MAP only increased by 18 ± 2% and 22 ± 2% respectively in response to these stimuli in lean CAT+ mice (*P*_Genotype_ ≤ 0.001; Figs 4 and 5). Feeding and shaker stress induced increases in systolic and diastolic arterial pressure were also less in CAT+ mice compared to WT mice (Table 2). Responses of HR and locomotor activity to restraint, shaker, and almond feeding stress did not significantly differ between lean WT and CAT+ mice (*P*_Genotype_ ≥ 0.12).

**Table 2:** Effects of stress on systolic and diastolic arterial pressure in lean mice.

	Δ SAP (mmHg)	Δ DAP (mmHg)
**Restraint**		
WT	26 ± 3	23 ± 3
CAT+	24 ± 2	21 ± 2
**Shaker**		
WT	28 ± 3	24 ± 2
CAT+	17 ± 2***	12 ± 1***
**Feeding**		
WT	27 ± 2	26 ± 2
CAT+	19 ± 2**	18 ± 2**

Data are mean ± SEM (*n* = 5–7).

** *P* ≤ 0.01

*** *P* ≤ 0.001 for CAT+ compared with WT mice

WT = wild type mice, CAT+ = endothelial CAT1 overexpressing mice, SAP = systolic arterial pressure, DAP = diastolic arterial pressure

### Responses to behavioural stress in obese mice

#### Restraint stress

The increase in MAP in response to restraint stress was less in obese WT mice (22 ± 2%), than lean WT mice (31 ± 3%; *P*_Adiposity within genotype_ = 0.02). In contrast, the increase in MAP during restraint stress was greater in obese CAT+ mice (37 ± 2%) than lean CAT+ mice (27 ± 2%; *P*_Adiposity within genotype_ = 0.01; *P*_Adiposity_*_Genotype_ < 0.001; Fig 3). In both genotypes of mice, obesity had minimal influence on increases in HR induced by restraint stress (*P*_Adiposity within genotype_ ≥ 0.60). As expected, locomotor activity ceased during restraint in all groups.

#### Shaker Stress

During 5 min of shaker stress, MAP increased by 32 ± 3% in lean WT mice and by 14 ± 2% in obese WT mice (*P*_Adiposity within genotype_ < 0.001). In contrast, responses of MAP to shaker stress did not significantly differ between lean and obese CAT+ mice (*P*_Adiposity within genotype_ = 0.48; *P*_Adiposity_*_Genotype_ < 0.001; Fig 4). The tachycardia induced by shaker stress was 35% less in obese CAT+ mice than in lean CAT+ mice (*P*_Adiposity within genotype_ = 0.05) but similar in obese WT mice compared to lean WT mice (Fig 4). Increases in locomotor activity induced by shaker stress were greater in obese WT (by 115%) and CAT+ mice (by 35%) than in lean WT and CAT+ mice (*P*_Adiposity_*_Genotype_ = 0.02; Fig 4).

#### Almond feeding

During almond feeding, MAP increased by 32 ± 2% in lean WT mice and by 20 ± 1% in obese WT mice (*P*_Adiposity within genotype_ < 0.001). In contrast, pressor responses to feeding did not significantly differ between lean and obese CAT+ mice (*P*_Adiposity within genotype_ = 0.28; *P*_Adiposity_*_Genotype_ < 0.001; Fig 5). The tachycardia induced by almond feeding was less in obese WT than in lean WT mice (*P*_Adiposity within genotype_ = 0.01), but similar in obese and lean CAT+ mice (*P*_Adiposity within genotype_ = 0.09; *P*_Adiposity_*_Genotype_ = 0.34). Locomotor activity during almond feeding was greater in obese CAT+ than in lean CAT+ mice (*P*_Adiposity within genotype_ = 0.03) but similar in obese and lean WT mice (Fig 5).

## Discussion

The novel finding in the current study was the effectiveness of endothelial specific CAT1 overexpression in neutralising the enhanced influence of the SNS on blood pressure in obesity, as demonstrated by the normalisation of the response to ganglionic blockade. We have previously shown that CAT+ mice have greater NO bioavailability compared to WT mice [4, 19]. NO can blunt the vasoconstrictor effects of the SNS within the periphery [8, 23] and can also reduce sympathetic outflow from the central nervous system [9]. It may therefore be that endothelial CAT1 overexpression and subsequent increases in NO bioavailability prevent obesity induced hypertension by blunting the influence of the SNS on blood pressure. Thus, increasing the bioavailability of NO may be a novel approach to attenuating the neurogenic hypertension associated with diet-induced obesity.

Endothelial CAT1 overexpression can prevent obesity-induced hypertension [4]. In the current study we examined whether the antihypertensive effects of CAT1 overexpression are dependent on the contribution of the autonomic nervous system to hypertension. We found that the depressor response to the ganglionic blocker pentolinium was augmented by fat feeding induced obesity in WT but not CAT+ mice. Consequently, the depressor response to pentolinium did not differ between obese and lean CAT+ mice and, importantly, MAP was similar in obese WT and CAT+ mice after ganglionic blockade. This provides evidence that obesity-induced hypertension in WT mice is driven, at least in part, by an increased dependence of basal MAP on autonomic influences. There is considerable evidence to support the view that obesity-induced hypertension in experimental animals and obesity-related hypertension in humans is due to increased activity of the SNS [24–26]. Augmentation of renal sympathetic nerve activity occurs very early in the development of obesity-induced hypertension [6]. Further, differences in MAP between fat-fed rabbits and those fed a normal diet are abolished by ganglion blockade [6].

Our previous [21, 27] and current data indicate that dirty cage stress, shaker stress, restraint stress and feeding can increase arterial pressure in lean WT mice. Three different stress stimuli were used in the present study because we hypothesised that, in obesity, pressor responses to stress are dependent on the type of stress stimulus [6, 14, 16]. Our current findings provide minimal support for this hypothesis as we found that pressor responses to both aversive and non aversive stress stimuli were reduced in obese WT mice. We previously found that obesity produced by a high fat diet reduced NO bioavailability in these mice [4]. We did not specifically quantify NO bioavailability within the brain in this study [4] but findings by others indicate that obesity can reduce central NO [12]. In this context, there is evidence that reductions in central NO can blunt pressor responses to stress [28]. Of note, we found that obesity significantly augmented pressor responses to restraint, but not shaker or feeding stress, in CAT+ mice. The precise mechanisms underlying the differential effects of CAT1 overexpression on pressor responses to stress remain to be determined. We previously found that CAT+ mice were resistant to obesity induced reductions in NO bioavailability [4]. Thus, it may be that CAT1 overexpression prevents reductions in NO, including that in the brain, in obesity, which in turn can enhance pressor responses to stress. This proposition is consistent with the ability of central NO to augment pressor responses to stress [28, 29].

Our previous data indicate that CAT+ mice have greater NO bioavailability compared to WT mice [4, 19]. Overexpression of CAT1 in our transgenic model is endothelial specific [19]. Consequently, we speculate that the impact of this transgene on NO bioavailability within the periphery is greater than that within the brain. Increased NO bioavailability within the periphery can blunt neurally mediated peripheral vasoconstriction during stress [30]. Consistent with this, we found that pressor responses to feeding and shaker stress were blunted in lean CAT+ mice compared to lean WT mice.

Our current data do not allow us to dissect out the relative effects of CAT1 overexpression on central *vs* peripheral NO bioavailability. As discussed above, NO bioavailability within the brain, and that within the periphery, appear to exert opposing effects on pressor responses to stress. This hypothesis merits investigation in the future.

We previously found that pressor responses to feeding and restraint stress were not significantly different between lean WT and CAT+ mice [22]. This is at odds with our present data which indicate that endothelial CAT1 overexpression can blunt pressor responses to feeding under normal physiological conditions. This discrepancy may relate to differences in stress exposure prior to assessing pressor responses to stress. In the present study, pressor responses to stress were assessed at the end of a 157 day diet protocol. In this protocol, mice were handled on a daily basis to examine any adverse effects of the diet and were weighed twice per week which involved briefly placing them in a small box. Consequently, this protocol may have exposed mice to chronic stress. Previously, pressor responses to stress stimuli were tested after infusion of diethyldithiocarbamic acid (DETCA) for only 11 days [22]. Thus, it may be that chronic stress impairs endothelial function and the ability of endothelial CAT1 overexpression to protect against pressor responses to feeding may only be revealed under these conditions. This is consistent with recent work which indicates that chronic exposure to noise stress can impair endothelial function in healthy adults [31].

Stress tests and pentolinium injections were performed/ administered during the periods when mice were inactive and active, respectively. This may explain the higher baseline blood pressure prior to pentolinium injections than that prior to stress tests. In addition, we performed experiments in freely moving conscious mice and consequently, blood pressure recordings were potentially confounded by feeding, drinking, locomotor activity and stress levels which may, at least in part, explain the differences in blood pressure during 1-h baseline recordings, immediately before stress tests and before pentolinium injections.

In conclusion, our data are consistent with the hypothesis that endothelial specific overexpression of CAT1 can prevent obesity induced hypertension, at least in part, by buffering the influence of the autonomic nervous system in regulating arterial pressure. Our findings also indicate that endothelial CAT1 overexpression can blunt pressor responses to feeding and shaker stress under normal physiological conditions. We conclude that treatment approaches which enhance L-arginine transport are likely to be beneficial under conditions of augmented SNS activity such as in obesity related hypertension.
